# Perspective: Barbie: Food for the Soul or Fanciful Nostalgia?^☆^

**DOI:** 10.1016/j.advnut.2024.100182

**Published:** 2024-02-01

**Authors:** Ellen S Rome

**Affiliations:** Center for Adolescent Medicine, Cleveland Clinic Children’s, Cleveland Clinic Lerner College of Medicine at Case, Cleveland, OH, United States

**Keywords:** Barbie, Barbie movie, body image, body positivity, empowerment, impact on youth, positive youth development

## Abstract

This perspectives piece analyzes the “Barbie” movie and its impact on its viewership. In contrast to prior research demonstrating that images of Barbie objectified girls, lowered self-esteem, and promoted body dysmorphia, with social media focus on “Fitspiration” as well as “Thinspiration” amplifying the negative effect, the Barbie movie may have more positive impact than one might predict. As gleaned from an informal survey of patients, parents, and peers, the messages of the Barbie movie include a mix of body positivity, recognition of the impact of depression and other aspects of mental health, critique of the perceived societal patriarchy, and a message of empowerment for girls, females, and people otherwise unrecognized.

**Statement of Significance**

Barbie has been criticized in social media and in formal research for her impossible figure, promoting body dysphoria despite her myriad career choices. Yet, the “Barbie” movie may move beyond the expected stereotypical response to allow for dark emotions to be expressed and hope to be within reach, and for the empowerment of girls, women, and others previously unrecognized.

When asked to write a commentary on the “Barbie” movie, my first response was to schedule a date with my Adolescent Medicine colleagues to view this cultural phenomenon. Second, I reflected on my own childhood relationship with Barbie, whom I confess, I owned and loved. And, when the American Academy of Pediatrics sent Dr Seuss books along with Barbie stories, I read all of those stories to my kids. The messages in each of the Barbie books and from Mattel were similar: Barbie could do and be anything, and she was a loyal friend, responsible older sister, a great doctor, a President, an astronaut, and any other career you could envision. In my youth, she had an impossible figure and was also blonde haired and blue eyed, as were most of the Disney princesses back then—and I must admit, for my first 2 decades, I always wished I had blonde hair and blue eyes, reflecting the power of imprinting. In the books and our childhood play, Barbie notably lacked parents, or any parental guidance, a fact that even my kids commented on at ages 4 and 6 years At their young ages and with my pediatric perspective, we deemed a flaw in the plot. So my historical lens reflected some of the social myths that Barbie perpetuated to this educated and not particularly sophisticated Barbie connoisseur. Barbie reflected and shaped society, but was she chicken or egg? What is her impact today and ongoing? Do we want her impacting our kids?

That brings us to the movie, which far exceeded any expectations I could have had. As with the original Barbie from Mattel, this movie was made to be a blockbuster and succeeds at every material turn. Directed by Greta Gerwig, this phenomenon has grossed over $1.4 billion globally since its release in the United States on July 21, 2023 [1]. With Margot Robbie as Barbie and Ryan Gosling as Ken plus an incredible list of supporting cast including America Ferrera, Kate McKinnon, Rhea Perlman, Will Ferrell, Issa Rae, Arianna Greenblatt, and others, Gerwig stacked the deck for both viewership and enjoyment. Barbie toy sales increased by 25% in the 2 months after its debut, as compared with the same 2 months in the prior year. On June 1, 2023, Mattel released a new collection of toys reflecting the movie characters plus a 3-story replica of the Barbie DreamHouse [2]. Mattel has solidified over 165 partnerships with brands and retailers just in time for the 2023 holiday season, extending its reach far beyond dolls and Ken’s Dojo Moja Casa House, with Barbie furniture, candles, and food sources, including Barbie burgers, pasta dishes, and Barbie-inspired sushi [2]. And, the impact on both big and little girls continues, with Barbie costumes outnumbering all other children’s costumes, only outperformed in the adolescent and adult market per TikTok search of “Travis Kelce and Taylor Swift costume,” which garnered nearly 427 million views. In contrast, “Barbie and Ken Halloween costume ideas” scored only 241 million views. Both Barbie and Taylor Swift are big business, role modeling what females can achieve while floating through life with flawless figures. Unlike Barbie in the movie or elsewhere, Taylor Swift has publicly spoken about her battle with body dysmorphia and disordered eating, as featured in the documentary “Miss Americana,” directed by Lana Wilson. She actively role models resiliency, singing, and speaking about her own vulnerabilities. In contrast, Barbie in the movie engages in body dysmorphic behavior, calling herself ugly and expressing fear and disgust over the potential gain of cellulite. Of note, Helen Mirrin, as movie narrator, calls out the discrepancy between the reality of Margot Robbie’s perfect Barbie-like figure and Barbie’s professed self-dislike and body dysmorphia. And, this movie, for the first time in Barbie’s history, acknowledges, albeit with humor, that depression, anxiety, and other mental health disorders exist as real emotions that many individuals must face. That said, does Barbie, like Taylor, provide more joy than body distress to young girls? Is the associated consumerism representative of money well spent? These are controversial questions that remain unanswered.

As one does in medicine, we can wrap the Barbie effects in a convenient theory. Social comparison theory suggests that people have a strong tendency to compare themselves with others in a quest for more accurate self-evaluation, drawing comparisons with those who are most similar to themselves and valuing themselves more or less through this comparison [3,4]. These social comparisons begin in early childhood, with young girls more likely to engage in these appearance-based comparisons [[4], [5], [6]]. These comparisons occur unconsciously and automatically, making us proceed with caution re-exposure to youth younger than 12 y of age or without attention to processing what they saw in a movie moment. An Australian study of 160 girls aged 5–8 years found that exposure to Barbie, whether in observation, active play, or on print media, correlated with more thin-ideal internalization but had no impact on body esteem or body dissatisfaction [7]. How does Curvie Barbie fit in? In 2016, Mattel made it to the front page of the “New York Times” with its release of the Fashionista Barbies with different sizes and proportions. And, by 2019, we had already found that girls were less likely to play with Plus Size Barbies [8]. In a study of 84 girls aged 3–10 years, Curvy Barbies provoked more negative attitudes and were identified as the dolls with whom they least likely wanted to play. And higher rates of body dissatisfaction correlated with more positive attitudes toward the original, slender, unrealistically proportioned Barbie. As the authors note, just having diverse Barbie shapes available did not overcome harmful weight stigma. When Barbie turned 50 in 2009, Worobey surveyed 254 college women about childhood Barbie play, family characteristics, body image satisfaction, and eating behaviors [9]. The author found that neither age of acquisition of Barbies or number of Barbies owned had a significant impact on self-evaluations of dieting behaviors or appearance. However, family’s value on physical appearance was the strongest predictor of dieting behavior. Again, Barbie as chicken or egg for the mother, impacting the daughter…?

Diving deeper into Barbie’s impact on body image, her perfect figure has been criticized as a model for Thinspiration so much that in 2016, in addition to debuting Curvy Barbie®, Mattel created Made to Move® dolls designed to encourage more active play among children [10,11]. Instead of “Thinspiration,” the goal of these dolls was more “Fitspiration” [10]. In addition to social comparison theory, objectification theory and sociocultural theory (the Tripartite Influence Model) were also invoked, noting that traditional or stereotypical Barbie personified a sexualized, appearance-potent, aspirational image that promoted unrealistic body proportions while undermining girls’ and women’s body image and self-esteem [3,[10], [11], [12], [13]]. The advent of Made to Move Barbie coincided with a Fitspiration movement on social media, which failed in its intention of getting young people to move, instead augmenting the ideal of the skinny White athlete, resulting in increased drive for weight loss, disordered eating and lower self-esteem for girls and women of color and with curvaceous figures [10,[14], [15], [16]]. In a recent study of 106 college women, Webb et al. [10] compared passive viewing of Barbie Fashionista dolls compared with Barbie Made to Move dolls compared with images of Lego Friends. Again, Barbie’s impact on self-esteem and body love was subtly subversive, even when she was supposed to be championing fitness. So, whether Barbie is a doctor, astronaut, yoga aficionado, or baseball star, when it comes to body image, it is still a strike out for Barbie.

Despite the potential for negative impact on self-esteem, our Adolescent Medicine team as well as all of our patients informally surveyed, along with their mothers, still came out Barbie-movie positive. For those who have not seen it yet, spoiler alert: watch it now. And, read on, so that you augment your appreciation for what this movie represents. There are some pieces and parts that were as expected from Barbie lore of childhood past. Barbie could still be anything, and the opening scene reveals Barbie awakening, getting out of bed and into her slippers with the high heels and then into the shower, with those heels still perpetually elevated, in the pretend Barbie house shower that has no actual water coming out. Each successive detail gave our Adolescent Medicine crew belly laughs. As happens in pretend play with Barbie, Barbie pretends to eat or eats invisible food—a detail amplified for our patients who restrict intake. Next, Barbie joyously greets each of her neighbor Barbies, who she can see from her house, all of whom are named—you guessed it—Barbie. More Barbie laughs. Then she floats down from her house into her Barbie car, exactly as a little girl might have moved her in real time play—again, belly laughs from us. We recognized that the proportions of things were a little non–life-like, but did not specifically realize that Barbie was made 23% bigger than her Barbie car and objects to reflect the fantasy of her world. And as for the heel cords, I spent 10 years of my life wearing 3 inch heels, during which time I served as an Associate Chief of Staff for the Cleveland Clinic and believed, consciously and unconsciously, that my extra height was more C suite worthy despite the fact that those 3-inch heels truly did shorten my Achilles’ tendons. This decade, I am stretching out those heel cords and raising my consciousness of choices that impact wellbeing as well as gravitas at work.

Even before I saw the movie, I started collecting quotes from my patients and their parents who had seen the movie. I heard repeatedly messages of body positivity and self-love, with the concept that you do not have to be perfect. I also heard loud and clear that you “can’t just be plastic,” and that “it’s not Ken’s world, but Ken can still be ‘Kenough.’” After I saw it, I heard their quotes with a more sensitized ear. I especially loved the response of one particular patient and his mother, an exceptional kid living in exceptional circumstances. This young person is living as a trans male, accepted by his family, school, and community, consistent with his persona from early childhood, per his parents. His mother shared the same thoughts reflected earlier about the movie emphasizing self-love. When I asked my 14-year old patient what he liked most, he sincerely replied, “I liked the mother daughter theme and how they learned to see and appreciate each other’s strengths.” Both his mother and I got a bit teary, and I especially loved how this mother and her now-name-legally-changed-son could share smiles and a hug over their own version of a mother–child journey. Together, we reflected on America Ferrera’s amazing speech, our favorite of the movie, where through her portrayal of Gloria, she is speaking to Barbie and, indirectly, to her daughter. I quote it now in its entirety, both for those who have and have not seen the movie:

It is literally impossible to be a woman. You are so beautiful and so smart, and it kills me that you don’t think you’re good enough. Like we have to always be extraordinary, but somehow we’re always doing it wrong.

You have to be thin, but not too thin. And you can never say you want to be thin. You have to say you want to be healthy, but also you have to be thin. You have to have money, but you can’t ask for money, because that’s crass. You have to be a boss, but you can’t be mean. You have to lead, but you can’t squash other people’s ideas.

You’re supposed to love being a mother, but don’t talk about your kids all the damn time. You have to be a career woman, but also always be looking out for other people. You have to answer for men’s bad behavior, which is insane, but if you point that out, you’re accused of complaining.

You’re supposed to stay pretty for men, but not so pretty that you tempt them too much or that you threaten other women because you’re supposed to be a part of the sisterhood. But always stand out and always be grateful. But never forget that the system is rigged. So find a way to acknowledge that but also always be grateful.

You have to never get old, never be rude, never show off, never be selfish, never fall down, never fail, never show fear, never get out of line. It’s too hard!

It’s too contradictory and nobody gives you a medal or says thank you! And it turns out in fact that not only are you doing everything wrong, but also everything is your fault.

I’m just so tired of watching myself and every single other woman tie herself into knots so that people will like us. And if all of that is also true for a doll just representing a woman, then I don’t even know.

—Gloria

There has been a ton of commentary on that speech, which I distill into this nugget: the Barbie movie has a Spine, or as writer and actress Shanee Edwards notes, a “central narrative that drives the plot and the character’s actions… a backbone that connects all the main events and actions of the characters as they explore and question the theme(s)” [17]. The themes, as you can imagine from what I addressed above, include self-acceptance and self-love, idealized femininity and unrealistic expectations, roles of males and females in society, and the impact of consumerism. Barbie’s journey to the real world to fix her fallen arches and new preoccupation with death meets with stark reality in the male-dominated world she finds, where her problems are not so easily solved. Gloria serves as the Mattel insider, but in a role of secretary rather than CEO. As a single mother, she mourns her daughter’s rejection of Barbie dolls and all that Gloria values from childhood; we feel Gloria’s pain over Sasha growing up too fast. We find that it is Gloria’s views of death and darkness that have infiltrated Barbie’s mindset. And Gloria believes that if she can help Barbie, she can build a bridge back to her daughter and a better world. By highlighting the cognitive dissonance that Barbie and her audience feel, that the world is not perfect in its current iteration, we can incite change to form a new world. There is hope in that.

The metaphor of having your feet planted firmly on the ground continues; Barbie first realizes that something is wrong with her when she realizes she now has flat feet. She immediately perceives this break with her Barbie world persona as a negative, amplified later by her response to newly perceived cellulite. When she decides to go to the real world to find the “cure,” with Ken stowing away in her Barbiemobile to accompany her, she encounters the misogyny of the real world. Although Ken finds the patriarchal, chauvinist attitudes kind of refreshing, Barbie notes that “It feels violent to me.” Barbie pushes back against being objectified. When another real-world male slaps Barbie’s butt without her consent, Barbie punches him in the face and is then arrested. This moment could plant the seed for later conversations with your tween or teen about boundaries and whether it is ever ok to have your butt grabbed, or to be the grabber, and what should be a proper response if that happens.

Reminder—the Barbie movie earned its PG-13 rating for “suggestive references and brief language.” One friend, father to 2 sons aged 9 and 11 years old was highly disappointed. He states, “As a dad who thinks a lot about the kind of men I want my sons to grow up to be, I was struck by the fact that a movie that spends so much time sending positive messages to young girls—and is widely praised for it—doesn’t seem to care a bit about the anti-male messages to young boys.” One Facebook friend of his had responded, “The fact that you noticed it was not pro-men is the point. No one ever notices that every other movie is not pro-women. Because that’s normal.” And another wrote, “Barbie is teasing men relentlessly. And we women laugh because every joke is spot on. The men who understand the teasing and get a kick out of it because they realize it’s done with affection are the ones the movie is talking ‘to.’ The ones who get angry are the ones the movie is talking about.” My answer is to engage in discussions about what his boys and he saw as flaws or spoof in the film, and what the real messages should be for all of us. As to the proper age to see this film? If parents bring their younglings to this film, this movie requires processing and discussion, especially for our littlest ones. This movie seems made for adults, especially ones with memories of our own childhood. But it can be a moment for thoughtful discussion—and belly laughs—with early and middle adolescents, who can appreciate all of its subtleties as well as be at risk of negative attitudes in the viewing. Dinner worthy conversation, for sure!

So back to our original question. Should we watch the Barbie movie with our kids? Does playing with Barbie harm their psyche? It seems that the context of how we parent, and encourage our patients and families to live and play, has more impact than any one doll. Our weighty talk at home may pile onto any negative self-image induced by play with an out of proportion doll. And the movie provides ample opportunity for connection, values clarification, and family discussion over dinner or otherwise. My 26-year-old son came away with 2 pearls. As he stated, “The message of the Barbie movie is about how everyone should take time to figure out who they are and what makes them happy, since they can’t live life trying to fit into the expectations of others.” That message warmed my heart, as he pursues his own dreams actively and continues to live life deeply, thoughtfully and authentically. And his second insight warmed my Adolescent Medicine self: “Every girl needs to see a gynecologist.” Slam dunk for warm handoffs to Adolescent Medicine!

## Conflict of interest

The author reports no conflicts of interest.

## Funding

The author reported no funding received for this study.
